# Regulation of cochlear hair cell function by intracellular calcium stores

**DOI:** 10.3389/fncel.2024.1484998

**Published:** 2024-11-25

**Authors:** Ghanshyam P. Sinha, Gregory I. Frolenkov

**Affiliations:** Department of Physiology, University of Kentucky, Lexington, KY, United States

**Keywords:** cochlea, deafness, mechanotransduction, electromotility, calcium signaling

## Abstract

**Introduction:**

Mammalian hearing depends on the dual mechanosensory and motor functions of cochlear hair cells. Both these functions may be regulated by Ca^2+^ release from intracellular stores. However, it is still unclear how exactly intracellular Ca^2+^ release may affect either hair cell mechano-electrical transduction (MET) or prestin-dependent electromotility in outer hair cells (OHCs).

**Methods:**

Here, we used photo-activatable (caged) compounds to generate fast increases of either Ca^2+^ or inositol-3-phosphate (IP_3_) in the cytosol of young postnatal rodent auditory hair cells, thereby stimulating either Ca^2+^- or IP_3_- induced releases of Ca^2+^ from intracellular stores. Fast Ca^2+^ imaging was used to monitor propagation of Ca^2+^ signals along the length of a hair cell. To access potential physiological role(s) of intracellular Ca^2+^ releases, we used whole cell patch clamp to record: i) OHC voltage-dependent capacitance, a known electrical correlate of prestin-based electromotility, and ii) MET currents evoked by stereocilia bundle deflections with fluid-jet. In the latter experiments, changes of mechanical stiffness of the hair bundles were also quantified from video recordings of stereocilia movements.

**Results:**

Ca^2+^ uncaging at the OHC apex initiated Ca^2+^ wave propagating to the base of the cell with subsequent Ca^2+^ build-up there. Ca^2+^ uncaging at the OHC base generated long-lasting and apparently self-sustained Ca^2+^ responses, further confirming Ca^2+^-induced Ca^2+^ release in the OHC basal region. Photoactivated IP_3_ initiated a slow increase of cytosolic Ca^2+^ ([Ca^2+^]_*i*_) throughout the whole OHC, confirming the presence of slow-activated IP_3_-gated Ca^2+^ stores in OHCs. Interestingly, Ca^2+^ uncaging produced no effects on OHC voltage-dependent capacitance. In an OHC, the rise of [Ca^2+^]_*i*_ is known to decrease axial stiffness of the cell and may modulate the stiffness of mechanosensory stereocilia bundles. To separate these two phenomena, we explored the potential effects of intracellular Ca^2+^ release on mechanical properties of stereocilia bundles in cochlear inner hair cells (IHCs). Ca^2+^ uncaging at the apex of an IHC caused a long-lasting increase in mechanical stiffness of stereocilia bundle without any changes in the amplitude or deflection sensitivity of the MET current.

**Discussion:**

We concluded that the most likely physiological role of IP_3_-gated Ca^2+^ release at the apex of the cell is the regulation of hair bundle stiffness. In contrast, Ca^2+^-induced Ca^2+^ release at the base of OHCs seems to regulate axial stiffness of the cells and its hyperpolarization in response to efferent stimuli, without direct effects on the OHC prestin-based membrane motors.

## Introduction

Although it is well-known that intracellular Ca^2+^ may regulate multiple processes in mammalian cochlear OHCs, the exact mechanisms of this regulation are still largely unknown. The major routes of entry of extracellular Ca^2+^ into an OHC include non-selective MET channels at the tips of mechanosensory stereocilia (Beurg et al., 2009), ATP-gated P2X receptors at the apex of the cell (Housley et al., 1998; Mammano et al., 1999), and acetylcholine-gated non-selective cation channels at the base of the cell (Evans et al., 2000). The mechanisms for extrusion of free Ca^2+^ out of the cell include plasma membrane calcium ATPase isoforms PMCA2 at the OHC stereocilia bundle (Chen et al., 2012; Dumont et al., 2001; Grati et al., 2006), PMCA1 at the basolateral plasma membrane of an OHC (Dumont et al., 2001; Grati et al., 2006), and Na^+^/Ca^2+^ exchanger at the OHC plasma membrane (Ikeda et al., 1992). A substantial amount of free cytosolic Ca^2+^ could be sequestered by intracellular Ca^2+^-buffering proteins that are expressed in the OHCs at extremely large concentrations (Hackney et al., 2005). A very dense mitochondrial network in the OHCs could also uptake intracellular free Ca^2+^, especially after mechanical overstimulation (Fettiplace and Nam, 2019).

Besides all mechanisms mentioned above, cytosolic free Ca^2+^ could be pumped into endoplasmic reticulum (ER) by calcium ATPases and released from the ER either through ryanodine receptors (RyRs) or inositol-1,4,5-trisphosphate (IP_3_) receptors (IP_3_Rs). There is evidence for the expression of both these Ca^2+^ release channels in cochlear OHCs (Frolenkov et al., 2000; Grant et al., 2006; Mammano et al., 1999). RyR is involved in an autocatalytic mechanism whereby [Ca^2+^]_*i*_ elevation induces Ca^2+^ release known as calcium induced calcium release (CICR) (Berridge et al., 2003). IP_3_Rs are also localized at the ER membrane and could be regulated by both IP_3_ and Ca^2+^ (Mikoshiba, 2007). However, in contrast to RyRs, IP_3_Rs could evoke Ca^2+^ release from the ER only in the presence of IP_3_ (Foskett et al., 2007).

Cochlear OHCs possess an unusual ER network that largely devoid the cell body and concentrated at the apex of the cell as lamellar and tubulovesicular structures of so-called Hensen’s body (Lim, 1986; Mammano et al., 1999), at the lateral surface of the cell as the system of flattened subsurface cisternae underlying cortical cytoskeleton (Furness and Hackney, 1990; Holley et al., 1992; Triffo et al., 2019), and at the base of the cell as synaptic cisternae near the plasma membrane (Fuchs et al., 2014). The lack of cytoskeletal components in the cell body seems to be a requirement for extremely fast length changes of OHCs known as electromotility (Brownell et al., 1985; Kachar et al., 1986), which are driven by voltage-dependent conformational changes of prestin molecules (Zheng et al., 2000) localized at the lateral plasma membrane with unusually high density (Belyantseva et al., 2000; He et al., 2010).

The components of the ER network in the cochlear OHCs were hypothesized to operate as intracellular Ca^2+^ stores. However, it is still not entirely clear how exactly the Ca^2+^ release from these hypothetical stores is activated and what might be the physiological function of such release. The best studied system is the synaptic cisternae at the base of OHCs. It is known that efferent neurotransmitter acetylcholine activates α9α10 nicotinic acetylcholine receptors (nAChR) at the base of OHCs, initiating local increase of [Ca^2+^]_*i*_, subsequent activation of Ca^2+^-activated potassium channels, and eventual hyperpolarization of the cell [reviewed in Fuchs and Lauer (2019)]. Pharmacological evidence indicates that this process is likely to be modulated by RyRs, i.e., involves CICR from the Ca^2+^ stores in the synaptic cisternae (Lioudyno et al., 2004). However, CICR at the base of an OHC has not been directly visualized.

Expression of intracellular Ca-ATPase in the OHC lateral wall (Schulte, 1993) and proximity of the lateral wall subsurface cisternae to the cortical cytoskeleton and prestin-rich plasma membrane suggest a possibility for regulation of electromotility and/or cortical cytoskeleton by Ca^2+^ release from presumable store in the subsurface cisternae. Indeed, Ca^2+^-dependent changes of the axial OHC stiffness are well documented following the application of acetylcholine or Ca^2+^ ionophores to isolated OHCs (Dallos et al., 1997; Frolenkov et al., 2003). There is also pharmacological evidence for the potential regulation of prestin function through Ca^2+^/calmodulin-dependent phosphorylation (Frolenkov et al., 2000; Frolenkov et al., 2001). Yet, Ca^2+^ release from this presumable store has never been visualized.

In contrast, it is known that OHCs possess IP_3_-gated Ca^2+^ store at the apex of the cell that could be activated by ATP-gated metabotropic P2Y receptors (Mammano et al., 1999). It was proposed that Ca^2+^ release from this store may modulate the mechanical stiffness of the stereocilia bundle. Indeed, some older experiments indicated that the hair bundle stiffness might increase upon [Ca^2+^]_*i*_ increase, but these experiments used global unphysiological Ca^2+^ changes produced by ionophores (Kossl et al., 1990; Pae and Saunders, 1994).

Here, we used high-speed Ca^2+^ imaging and photo-activatable (caged) compounds to generate fast increases of either Ca^2+^ or IP_3_ in the cytosol of young postnatal rodent auditory hair cells and explore the resulting release of free Ca^2+^ from different putative stores along the OHC axis. We also examined the direct effects of photo-activated Ca^2+^ on prestin activity and mechanical stiffness of the stereocilia bundle. Our data demonstrate the presence of CICR at the base but not the apex of an OHC, lack of direct Ca^2+^ effects on prestin activity, and the increase of stereocilia bundle stiffness upon rise in [Ca^2+^]_*i*_.

## Materials and methods

### Cell preparation

**FIGURE 1:**
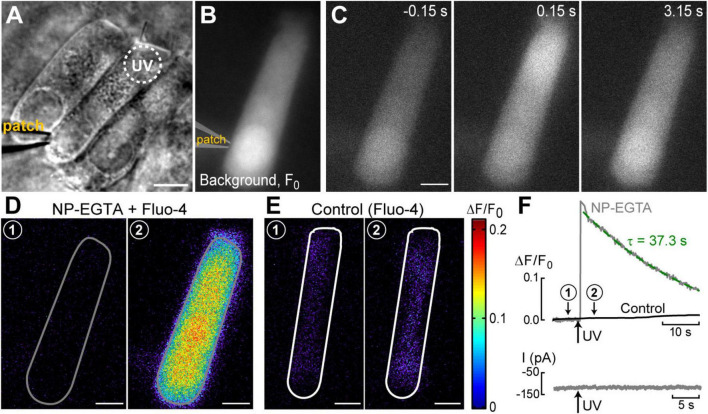
UV photolysis of photo-activable (caged) Ca^2+^ chelator releases free Ca^2+^ in cochlear outer hair cells (OHCs). **(A)** Bright field image of the organ of Corti explant with an OHC patched with a pipette containing 1 mM of NP-EGTA pre-mixed with 0.7 mM of Ca^2+^, 100 μM of Fluo-4, and 147 mM of Cs^+^. Dotted circle shows the region of UV illumination. **(B)** Epi-fluorescent image of another OHC loaded with NP-EGTA+Ca^2+^+Fluo-4. This image was obtained by averaging all frames in a time-lapse sequence before UV illumination and used as a reference (F_0_) image. **(C)** Individual images from this time-lapse sequence collected before (negative time stamp) and after (positive time stamps) UV illumination. **(D)** Individual ΔF/F_0_ ratio images from the same time-lapse sequence in pseudocolor scale before (1) and after (2) UV illumination. **(E)** Similar pair of ΔF/F_0_ ratio images from a time-lapse sequence in a control cell that was loaded only with Fluo-4 (no caged Ca^2+^). **(F)** Top: traces of the changes in normalized (ΔF/F_0_) fluorescence intensity over time in an OHC loaded with “caged Ca^2+^” (NP-EGTA+Ca^2+^, gray) and in a control OHC loaded only with Ca^2+^ indicator, Fluo-4 (black). The data are from the same cells that are shown in **(D,E)**. The average intensity of fluorescence was calculated in region of interest outlying the whole cell (gray and white lines in **(D,E)**, correspondingly). Arrow indicates the timing of UV flash illumination. Numbers point to the timing of images shown in **(D,E)**. The decay of fluorescence after UV flash in a cell loaded with caged Ca^2+^ fits to an exponential curve with a time constant of τ = 37.3 s (dashed line). Bottom: simultaneous recordings of the whole-cell currents. Scale bars in all images: 5 μm. OHCs are from P10–P12 rats.

**FIGURE 2:**
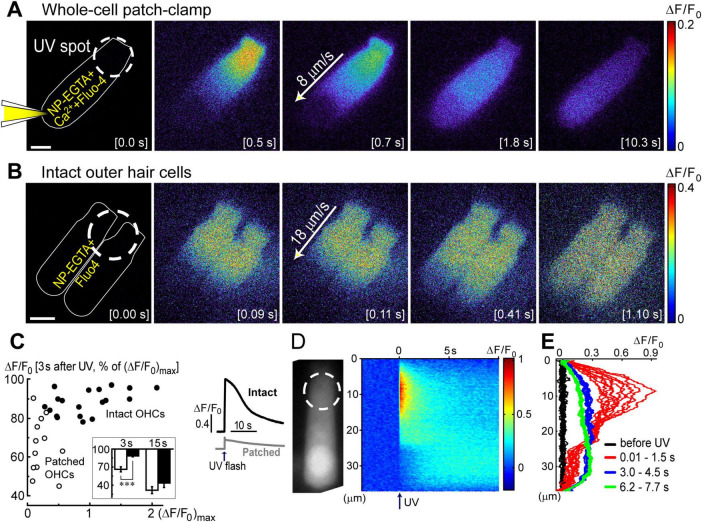
Ca^2+^ imaging reveals the presence Ca^2+^-induced Ca^2+^-release at the base of OHCs. **(A)** Time lapse sequence of the normalized changes in Ca^2+^ indicator fluorescence (ΔF/F_0_) after UV flash pointed to the apex of an OHC at the time point of 0.0 s (shown in the brackets at each image). Left cartoon indicates that the cell was loaded with NP-EGTA+Ca^2+^+Fluo-4 through the pipette in a whole-cell patch-clamp configuration. Wide-field epi-fluorescent imaging. **(B)** Similar time lapse imaging but in an intact unpatched OHC loaded with cell permeable NP-EGTA and Fluo-4 and recorded with a spinning disk confocal unit to avoid out-of-focus fluorescence. Arrows in the middle images indicate the speed of Ca^2+^ signal propagation along the length of an OHC. Scale bars: 5 μm. **(C)** Left: scattergram of the percentage of the remaining Ca^2+^ response at 3 s after UV flash relative to the maximal response (ΔF/F_0_)_*max*_ in the same OHC. Cells in whole-cell configuration are shown with open symbols, while intact non-patched cells are shown with closed symbols. Inset shows average percentages of the remaining Ca^2+^ response at 3 and 15 s following UV flash in patched (filled bars) and intact (open bars) OHCs. Asterisks show statistical significance: ***, *p* < 0.001 (Student’s *t*-test). Right: representative traces of the average Ca^2+^ signal from the total area of intact (black) or patched (gray) OHCs. The arrow indicates the timing of the UV flash. **(D)** Left: non-normalized baseline image of Fluo-4 fluorescence in a patched OHC. Dashed circle shows the location of the UV laser spot. Right: rastergram of normalized Ca^2+^ response along the length of this OHC (*Y*-axis) at different times (*X*-axis) before and after UV flash at time point 0.0 s (arrow). **(E)** Selected intensity profiles of Ca^2+^ response along the longitudinal axis of the OHC in (D) at four different time windows (indicated in the legend). All data are from rat P10–12 OHCs, except panel C that combines rat P10–12 OHCs and mouse P8 OHCs.

**FIGURE 3:**
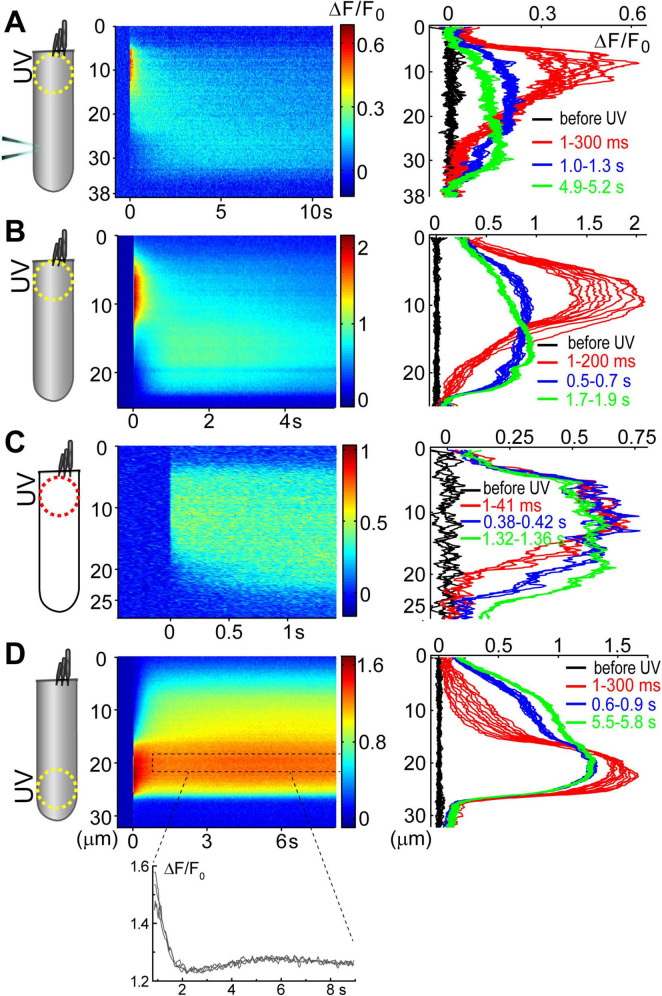
Ca^2+^ build-up at the basal region of the OHCs at different experimental conditions. All panels show a schematic cartoon of experimental conditions on the left panels, a rastergram of the normalized Ca^2+^ response along the length of an OHC on middle panels, and selected intensity profiles of the Ca^2+^ response along the longitudinal axis of an OHC at different time windows on right panels (see Figures 2D, E for more details). Patch pipettes in the cartoons indicate whole-cell patch-clamp conditions. The lack of intracellular shading on the cartoon indicates confocal imaging. **(A)** Ca^2+^ uncaging at the apex of a patched OHC. **(B)** Ca^2+^ uncaging at the apex of an intact unpatched OHC. **(C)** Short-term fast confocal imaging of an intact OHC after Ca^2+^ uncaging at the apex. **(D)** Ca^2+^ uncaging at the base of an intact OHC. Bottom inset shows ΔF/F_0_ changes over time from a dashed region in the rastergram, demonstrating the secondary peak of Ca^2+^ response.

**FIGURE 4:**
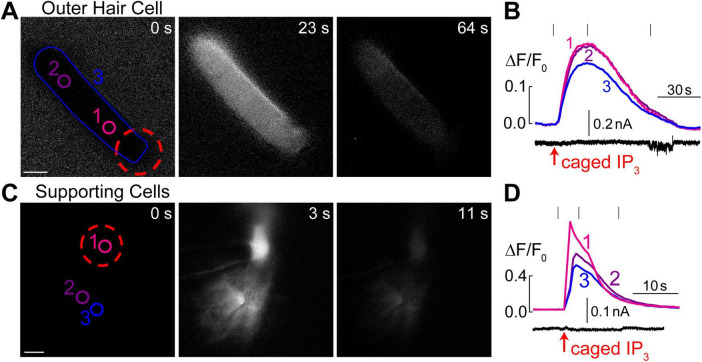
Intracellular Ca^2+^ stores in OHCs are gated by IP_3_. **(A)** Time-lapse images of normalized Ca^2+^ responses (ΔF/F_0_) in an OHC loaded through a patch pipette with caged IP_3_ and Fluo-4 before UV flash (0 s), during maximal Ca^2+^ response (23 s), and after recovery (64 s). **(B)** Top, measured Ca^2+^ responses in the regions of interest indicated in panel A: #1–OHC apex, #2–base of the OHC, and #1–entire OHC. Bottom, simultaneous recording of the whole cell current at a holding potential of –60 mV. **(C)** Similar time-lapse images of normalized Ca^2+^ responses but in supporting cells located near the third row of OHCs and loaded through a patch pipette containing caged IP_3_ and Fluo-4. Due to gap junction conductance, caged IP_3_ and Fluo-4 diffuse not only to the cell that was patched (#1) but also into the neighboring cells (#2 and #3). **(D)** Similar to panel B, Ca^2+^ responses in the numbered regions of interest are shown together with simultaneous recording of the whole-cell current in cell #1 at a holding potential of –20 mV. Scale bars: 5 μm. All data are from rat organ of Corti explants at P7–P11.

**FIGURE 5:**
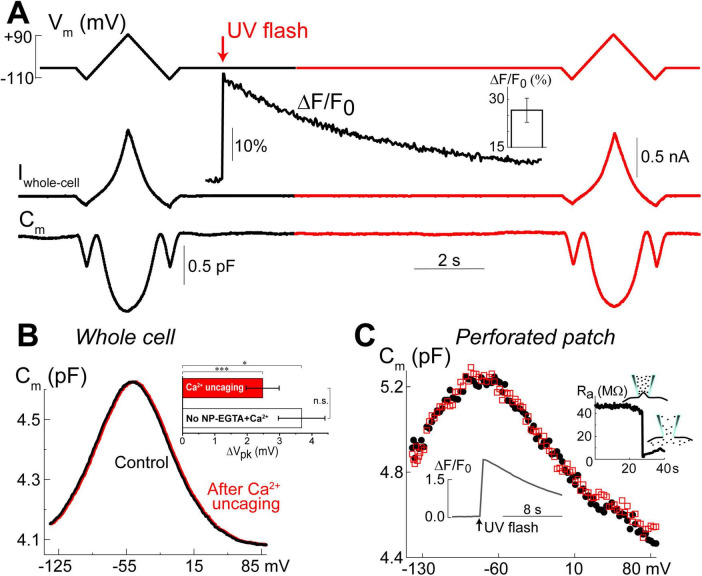
Ca^2+^ uncaging does not affect non-linear capacitance in OHCs. **(A)** Simultaneous recordings of holding potential (V_m_, top), whole-cell current (I_whole–cell_, middle), and membrane capacitance (C_m_, bottom) in an OHC before and after Ca^2+^ uncaging (arrow) that generated an increase of Fluo-4 fluorescence (inset trace). Note that the changes of C_m_ produced by voltage ramps from –110 mV to +90 mV before and after the increase of [Ca^2+^]_i_ are nearly identical. Holding potential between the ramps was –60 mV. This OHC is a representative of *n* = 14 OHCs that, on average, showed a 26.6 ± 3.8% increase of Ca^2+^ signal after UV-flash (Mean ± SE, bar graph in the inset). **(B)** OHC capacitance as a function of membrane potential derived from data in **(A)** before (black) and after (red) Ca^2+^ uncaging. Inset shows statistically significant but similar rightward drifts of C_m_(V) peaks in both, OHCs loaded with NP-EGTA+Ca^2+^+Fluo-4 (*n* = 14) and OHCs loaded only with Fluo-4 (*n* = 3, no Ca^2+^ uncaging). Asterisks show statistical significance of the changes of C_m_(V) peak position in the same cells (*p < 0.05; ***p < 0.001, paired *t*-test) or between the groups (n.s., non-significant, *t*-test). All other parameters of the C_m_(V) fit to the first derivative of a two-state Boltzmann function showed non-significant changes after Ca^2+^ uncaging (see Table 3). **(C)** Nearly identical C_m_(V) before (black solid circles) and after (red open squares) Ca^2+^ uncaging in another OHC but in gramicidin-mediated (120 μM) perforated-patch whole-cell recordings. Right inset: breaking of the membrane patch at the end of the experiment confirmed perforated patch conditions. Bottom inset: Ca^2+^ increase produced by UV flash in this OHC loaded with cell-permeable NP-EGTA+Fluo-4. All data are from mouse P8 OHCs.

**FIGURE 6:**
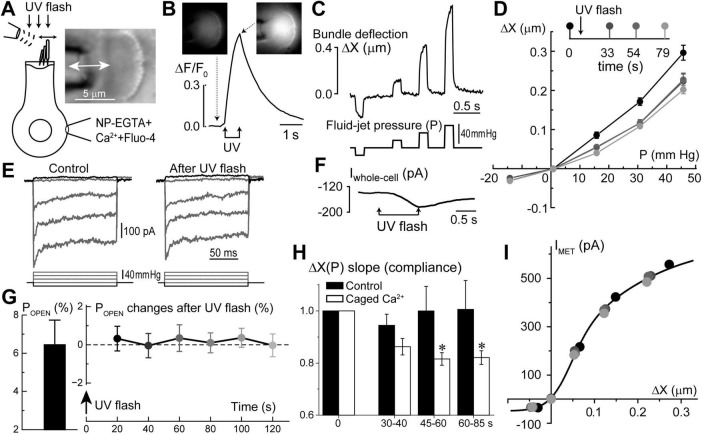
Long-term increase of hair bundle stiffness without apparent changes of mechano-electrical transduction (MET) after Ca^2+^ uncaging in the inner hair cells (IHCs). **(A)** Schematics of whole-cell patch-clamp recordings of MET currents evoked by fluid-jet with bright field image of an IHC stereocilia bundle (right inset). **(B)** Normalized (ΔF/F_0_) changes in Fluo-4 signal produced by UV flash focused on this IHC. Top insets show epi-fluorescent images of the cell before and after Ca^2+^ uncaging at the times indicated by arrows. To obtain a significant rise in [Ca^2+^]_i_, UV flash consisted of 150 pulses of 1 ms each over 750 ms duration. **(C)** Stereocilia bundle deflections (ΔX, top) produced by fluid-jet pressure stimuli (P, mm Hg, bottom). **(D)** Bundle deflection vs. fluid-jet pressure relationships at different time points (shown as different gradations of gray) before and after Ca^2+^ uncaging. **(E)** MET responses to step-like deflections of the hair bundle before and 33 s after Ca^2+^ uncaging. **(F)** Changes of the whole-cell current evoked by Ca^2+^ uncaging. Holding potential: –60 mV. All data shown in panels A through F were obtained from the same IHC. **(G)** Average value of the MET channel open probability at resting bundle position (P_OPEN_, left) in a group of IHCs (*n* = 9) and the changes of P_OPEN_ in this group with time after Ca^2+^ uncaging (right). Data are shown as mean ± SE. The changes of P_OPEN_ are not statistically significant (one-way ANOVA). **(H)** Changes of the hair bundle compliance (reciprocal to the stiffness) estimated from the slope of ΔX(P) relationship at positive bundle deflections in the same group of IHCs that is shown in panel G (*n* = 9). In each cell, compliance values were normalized to the ones obtained before Ca^2+^ uncaging (open bars). For comparison, black bars show the result of the same experiments, but in control IHCs loaded only with Fluo-4 but without NP-EGTA+Ca^2+^ (*n* = 4). Data are shown as mean ± SE. The difference between “Ca^2+^ uncaging” and “control” groups is highly significant (*p* = 0.00016, two-way ANOVA). Asterisks show statistical significance of post-hoc evaluation of changes at individual time points by *t*-test: 45–60 s, *p* = 0.023; and 60–85 s, *p* = 0.042. **(I)** MET current dependence on hair bundle deflection remains unchanged after Ca^2+^ uncaging despite changes in stiffness. The same cell as in panels (**A–F**). Line shows double-Boltzmann fit to the data measured at different time points relative to Ca^2+^ uncaging. All data of the figure are from rat IHCs at P4–P7.

With only one exception mentioned below, the organ of Corti explants were dissected from young postnatal rats or wildtype (C57BL6/J) mice at postnatal days 6–12 (P6–12). These are the ages when the OHC electromotility is already easily detectable (Belyantseva et al., 2000). We started this study using both rats and mice, hoping to find evidence for Ca^2+^-dependent regulation of OHC non-linear capacitance (NLC) and continue this study in specific mouse mutants. However, we also wanted to include rat hair cells since they are bigger and better suited for our imaging experiments. Therefore, all experiments with Ca^2+^-induced changes in OHC non-linear capacitance (Figure 5) were performed in mouse OHCs, while the studies of CICR (Figures 1–3) used both mouse and rat OHCs. No differences between the species were found in the latter experiments and the data were combined. After getting no Ca^2+^ effects on NLC, we continued IP_3_ uncaging (Figure 4) and stereocilia bundle stiffness (Figure 6) experiments only in rat hair cells at P7–P11 and P4–P7, correspondingly.

The most apical and the most basal (“hook”) regions were cut off before placing the explants in the glass-bottom Petri dishes, where they were held by pre-glued fine glass fibers. The Petri dish was filled with Leibowitz cell culture medium (L-15, Invitrogen) containing the following inorganic salts (in mM): 137.93 NaCl; 5.33 KCl; 1.26 CaCl_2_; 0.99 MgCl_2_; 1.33 Na_2_HPO_4_; 0.44 KH_2_PO_4_; 0.81 MgSO_4_. All experiments were performed at room temperature (22–24°C) in the cells located approximately in the middle of the cochlea. All animal procedures were approved by the University of Kentucky Animal Care and Use Committee.

### Patch-clamp recordings

Hair cells were observed using an upright Olympus BX51W1 microscope (Olympus, Center Valley, PA, USA) equipped with a 100× 1.0 N.A. water immersion objective. To access the basolateral plasma membrane of the OHCs and IHCs, a hole in the sensory epithelium was made by suction of a few outermost cells, next to the cell of interest. Then, a new fresh pipette filled with the bath L-15 medium was used to apply positive pressure around the selected cell to prepare a clean surface for patching. Finally, another pipette with an intracellular solution was used to establish whole-cell patch-clamp recordings. For conventional recordings, the pipettes were filled with our standard intracellular solution containing (in mM): CsCl (140), MgCl_2_ (2.5), Na_2_ATP (2.5), EGTA (1.0), HEPES (5). For experiments with caged Ca^2+^ chelators, the intracellular solution contained (in mM): CsCl (143.2), CaCl_2_ (0.7), NP-EGTA (1) and HEPES (10) supplemented with cell-impermeant Ca^2+^ indicator Fluo-4 (0.1) (Invitrogen / Molecular Probes). We found these relative concentrations of NP-EGTA and Ca^2+^ (1 mM vs. 0.7 mM, correspondingly) most effective based on minimal Fluo-4 fluorescence before UV stimulation and a large change in Fluo-4 signal after UV flash. For caged-IP_3_ experiments, the intrapipette solution contained (in mM): KCl (12.6), KGlu (131.4), MgCl_2_ (2.0), EGTA (0.5), K_2_HPO_4_ (8.0), KH_2_PO_4_ (2.0), Mg_2_ATP (2.0), Na_4_GTP (0.2) plus 100 μM of caged-IP_3_ (Enzo Life sciences) and 100 μM of Fluo-4. In all cases, the osmolarity of intrapipette solutions was adjusted with D-glucose to match corresponding values of L-15 (∼325 mOsm) and pH was adjusted to 7.3–7.4. The patch pipettes had a typical resistance of 3–5 MΩ in the bath. Patch-clamp recordings were performed either with the MultiClamp 700B computer-controlled amplifier (Molecular Devices) or with the optical feedback OptoPatch amplifier that has a build-in hardware-based tracking of cell capacitance (Cairn Research, Kent, UK). In both cases, data acquisition was controlled by pClamp 10 software (Molecular Devices). Between the records, HCs were maintained at a holding potential of −60 mV. No correction was applied for liquid-junction potentials or series resistance. During MET recordings, the holding potential was temporarily changed to −80 mV.

### Loading of the cells with cell-permeable compounds

Cell-permeable (AM) versions of NP-EGTA and Fluo-4 (both from Invitrogen) were dissolved in DMSO to obtain each stock solution of 4 mM concentration. Immediately before the experiment, the stock solution was diluted to 20 μM in DMEM supplemented with 250 μM of sulfinpyrazone. All specimens were incubated in the prepared solutions for 40 min at 37°C. The specimen was rinsed three times with fresh L-15 and used immediately for the experiment.

### Ca^2+^ imaging combined with flash-photolysis of caged Ca^2+^ chelators

Specimens were observed using an upright Olympus BX51W1 microscope equipped with a 100× 1.0 N.A. water immersion objective and DSU spinning disk confocal unit (Olympus, Center Valley, PA, USA). Before starting the experiment, a bright field image of the cell was examined for the signs of membrane blebbing, swelling of the cell body, and/or excessive movements of intracellular organelles. Only the cells without these signs of deterioration were used. The Fluo-4 fluorescence was excited by a mercury light source illumination passing through a 470/40 nm (center/bandwidth) filter and observed with a 525/50 nm filter. The illumination intensity was always adjusted to minimize photo-bleaching. Images were acquired with the Evolve 512 camera (Photometrics, Tucson, AZ) and MetaMorph software (Molecular Devices). Fast photo release of free Ca^2+^ was achieved by ultraviolet (UV) illumination generated by DPSL355/30 laser (Rapp OptoElectronic, Hamburg, Germany) that was coupled to the microscope through an optical fiber. The 355 nm laser beam was focused to a diameter of ∼5 μm at the specimen. It allowed directing the uncaging UV beam to a particular location within a hair cell that, in most cases, was oriented horizontally and perpendicularly to the beam. The UV light was delivered in pulses of 5–150 ms duration. To account for spatial variations in the total amount of Fluo-4 dye along and within the hair cell, changes in Ca^2+^ signal over time were normalized to the fluorescence before UV illumination (ΔF/F_0_) at each pixel. These traces were averaged to obtain the Ca^2+^ signal from an overall cell or a region of interest within a cell. The decay time constants were obtained by fit to a single-exponential curve at the first part of ΔF/F_0_ trace after UV flash. To explore the propagation of Ca^2+^ wave along the longitudinal axis of an OHC, two-dimensional plots were constructed to show the changes of a longitudinal profile of ΔF/F_0_ signal at different time points.

### Perforated-patch recordings

Perforated-patch recordings were performed in cells loaded with cell-permeable NP-EGTA and Fluo-4. The intrapipette solution additionally contained freshly prepared 120 μM of gramicidin. It took approximately 10–20 min for gramicidin to perforate the membrane. At the end of the perforated-patch experiment, we always broke the patch with negative suction through the pipette and monitored patch clamp parameters and Ca^2+^ fluorescence. Breaking the seal resulted in a decrease of series resistance without significant changes in cell capacitance readings, accompanied by a reduction of Ca^2+^ fluorescence, indicating effective diffusion of Ca^2+^ dye from a cell into the pipette.

### Capacitance measurements

Cell capacitance was measured with an Optopatch amplifier in the track-in mode. A 10-mV peak-to-peak sinus wave at 2.5 kHz was superimposed on a normal holding potential of −60 mV, and whole-cell compensation controls for series resistance and cell capacitance were manually adjusted to minimize the sinusoidal component of the whole-cell current. At this point, the built-in lock-in amplifier was turned on, the phase was manually optimized, and the capacitance and resistance dithering circuits were activated to calibrate the system. Rs values typically ranged from 5 to 12 MOhm. Cells displaying higher Rs values were discarded. The track-in feedback circuit was then switched on, and its gain gradually increased to the highest stable value (usually 50). C_*m*_ traces were recorded at 1 kHz and filtered online at 20 Hz. Slow ramps of the holding potential from −110 mV to +90 mV were applied to obtain the voltage-dependence of an OHC capacitance. The recorded capacitance was fitted to the first derivative of a two-state Boltzmann function as follows:

*C*_*m*_ = *C*_*v*_ + *C*_*lin*_, where *C*_*m*_ is the total membrane capacitance, *C*_*v*_ is a voltage-dependent (non-linear) component, and *C*_*lin*_ is a voltage-independent (linear) component.


$$C_{V}=Q_{m\,a\,x}\,\frac{z\,e}{k\,T}\,\frac{b}{{\left(1+b\right)}^{2}};b=e\,x\,p\,\left(\frac{-z\,e\,\left(V-V_{p\,k}\right)}{k\,T}\right)$$

where *Q*_*max*_ is the maximum nonlinear charge moved, *V*_*pk*_ is a voltage at peak capacitance, *V* is membrane potential, *z* is valence, *e* is electron charge, *k* is Boltzmann’s constant, and *T* is absolute temperature.

### Ca^2+^-induced changes of stereocilia bundle stiffness

To avoid artifacts of Ca^2+^-dependent changes of overall cell stiffness and associated “slow” motility in OHCs (Dulon et al., 1990; Frolenkov et al., 2003), the effects of intracellular free Ca^2+^ on stereocilia bundle stiffness were studied in cochlear inner hair cells (IHCs), which provided an additional benefit of larger stereocilia that are easier to track with automated image-processing algorithms. All IHCs were patched with our standard intrapipette solution supplemented with cell-impermeable NP-EGTA and Fluo-4. The stereocilia bundles were deflected with a fluid-jet driven by a first-generation analog high-speed pressure clamp (ALA Scientific, Farmingdale, NY, USA). Simultaneously with recordings of the mechano-electrical transduction (MET) currents, the deflections of hair bundles were recorded with an Evolve 512 camera. The digitized video images were analyzed off-line using routines developed with the Image Processing Toolbox of MATLAB (The Mathworks Inc.). The movement quantification was based on the modified algorithm previously developed to quantify OHC electromotility (Frolenkov et al., 1997). Briefly, we calculated a frame-by-frame shift of the intensity profiles along a line perpendicular to the stereocilia rows in a hair bundle. These measurements were repeated over three-to-five locations along the stereocilia row and averaged to obtain the average movement of the stereocilia bundle in this cell. Care was taken to include both central and side stereocilia in the bundle, except the very edges of the bundle. Then, the relationship between bundle deflection (ΔX) and fluid-jet pressure (P) was constructed for each cell before and after Ca^2+^ uncaging. Although the exact stiffness of the hair bundle cannot be determined with fluid-jet stimulation, the inverse of the slope of ΔX(P) relationship is proportional to the stiffness and the changes of this parameter in the same cell could be used as a measure of relative changes in the stereocilia bundle stiffness. The MET currents during these deflections were simultaneously recorded and the current-displacement I(ΔX) curves were constructed. These I(ΔX) curves were fitted to a second-order Boltzmann function to obtain the estimate of the open probability of MET channels at resting bundle position (P_*OPEN*_) as previously described (Peng et al., 2016).

### Statistical analysis

Most experiments required simple assessment of the differences between two groups of the cells or assessment of the changes happening before and after a single treatment (UV-flash). Correspondingly, two-sample or paired-sample *t*-tests with Welch correction were used. However, statistical significance of the changes of hair bundle stiffness over time and relative to control (Figure 6H) was estimated by two-way ANOVA with post-hoc assessment by *t*-test. All statistical analysis was performed with OriginPro 2023b (OriginLab Corporation, Northampton, MA, USA).

## Results

The first goal of this study was to visualize Ca^2+^-induced Ca^2+^-release (CICR) in cochlear OHCs. Although immunolabeling does show expression of RyRs in OHCs (Grant et al., 2006) and pharmacological studies indicated that CICR is likely to be functional in OHCs, at least at their base (Lioudyno et al., 2004), this phenomenon has not been yet demonstrated in OHCs directly by Ca^2+^ imaging. For this purpose, a very fast and localized increase of [Ca^2+^]_*i*_ is needed. Therefore, we used a patch clamp pipette to deliver into the hair cell a photo-activatable (caged) Ca^2+^ chelator NP-EGTA pre-loaded with Ca^2+^ and supplemented with a Fluo-4 Ca^2+^ indicator. After establishing whole-cell configuration and waiting for ∼5 min for drug diffusion into the OHC, we delivered a single short pulse of UV light collimated into ∼5 μm diameter to the cell (Figure 1A). UV illumination dramatically decreases the affinity of NP-EGTA to Ca^2+^ resulting in a large release of free Ca^2+^ (Figures 1B, C). This rise of [Ca^2+^]_*i*_ occurred in OHCs loaded with NP-EGTA+Ca^2+^ but not in control OHCs without caged Ca^2+^ (Figures 1D, E). The Fluo-4 signal raised within less than 2 ms (the fastest frame rate of the camera capturing fluorescent images in our experiments) and started to decay almost immediately after UV flash (Figure 1F, top). Although it was possible to increase the rise in Fluo-4 signal with repetitive UV pulses, the OHCs often became leaky, with the sharp drop in whole-cell currents and rise in Fluo-4 fluorescence, both of which did not return to baseline. We interpreted these non-recoverable changes after UV flash as cell damage due to Ca^2+^ overload and rejected such cells for further analysis. It should be noted that our standard intrapipette solution was based on CsCl rather than KCl and, therefore, the outward currents through Ca^2+^-activated K^+^ channels were blocked, resulting in no changes of whole-cell current upon UV stimulation in an undamaged OHC (Figure 1F, bottom). On average, Ca^2+^ uncaging produced ∼20% rise of Fluo-4 fluorescence in patched OHCs (Table 1) with only a small drop in whole-cell current by −9 ± 2 pA (changes are not significant as assessed by paired *t*-test). Thus, we developed experimental conditions for an abrupt increase of free Ca^2+^ inside OHCs without their damage and with the ability to study their function with whole-cell patch clamp.

**TABLE 1:** Parameters of Fluo-4 responses (Mean ± SE) produced by Ca^2+^ uncaging in OHCs in whole-cell patch clamp conditions and intact OHCs loaded with cell-permeant versions of NP-EGTA and Fluo-4.

	Whole-cell patch clamp	Intact unpatched cells
% change in fluorescence (ΔF/F_o_)	19.7 ± 3.5 (*n* = 18)	69.5 ± 13.0*** (*n* = 20)
Time constant of ΔF/F_o_ decay (τ, s)	10.0 ± 2.0 (*n* = 18)	14.4 ± 3.3^#^ (*n* = 20)
ΔI_UV_ (pA)	−9 ± 2 (*n* = 14)	–

^#^Fit was performed at the decay phase of Fluo-4 signal traces.

***p < 0.001, *t*-test with Welch correction.

### Propagation of UV-flash-induced Ca^2+^ signals is different in patched and intact OHCs

Next, we systematically investigated the propagation of Ca^2+^ signals along the length of an OHC. Two fundamentally different conditions of Ca^2+^ buffering inside an OHC were studied. First, the cell was loaded with NP-EGTA+Ca^2+^+Fluo-4 via patch pipette simultaneously with whole-cell recordings (Figure 2A). In this case, Ca^2+^ buffering is controlled by intrapipette solution and imaging does not require confocal mode since the recorded cell is the only fluorescent cell in the organ of Corti explant. In the second set of experiments, cochlear explants were loaded with cell-permeable (AM) versions of NP-EGTA and Fluo-4 before imaging (Figure 2B). In the latter case, the actual concentrations of NP-EGTA, Ca^2+^ bound to NP-EGTA, and Ca^2+^-sensitive portion of Fluo-4 inside the cell were all unknown but they are likely to be significantly less compared to the loading through patch pipette. Therefore, Ca^2+^ buffering inside an OHC was closer to the endogenous buffering. However, Ca^2+^ imaging sometimes required confocal mode due to the background fluorescence of other cells within the organ of Corti.

In both patched and intact OHCs, spot UV illumination to the apex of the cell produced an almost instant increase of [Ca^2+^]_*i*_ at the illumination site (Figures 2A, B). Then, Ca^2+^ signal propagated along the OHC longitudinal axis as a wave in case of a patched OHC (Figure 2A) and as a fast Ca^2+^ spread in case of an intact OHC (Figure 2B). The amplitude of UV-flash-induced increase in Ca^2+^ signal was significantly larger and the speed of apex-to-base propagation of this signal was considerably faster in intact OHCs compared to patched OHCs, while the difference in time constants of fluorescent signal decay was not statistically significant (Table 2). These results indicate apparently different Ca^2+^ buffering conditions that may affect the amplitude and spatial propagation of Ca^2+^ signals but similar Ca^2+^ extrusion mechanisms that are likely to determine the time constant of [Ca^2+^]_*i*_ clearance.

**TABLE 2:** Propagation speeds of Ca^2+^ signals along the longitudinal axis of OHCs (Mean ± SE).

	Whole-cell patch clamp	Intact unpatched cells
Propagation speed: apex-to-base (μm/s)	5.6 ± 0.6 (*n* = 7)	16.7 ± 1.2*** (*n* = 13)
Propagation speed: middle-to-base (μm/s)	8.9 ± 1.0* (*n* = 6)	–
Propagation speed: base-to-apex (μm/s)	11.9 ± 1.6* (*n* = 3)	19.5 ± 3.0 (*n* = 5)

***Statistical significance in apex-to-base propagation speed between patched and intact cells, *p* < 0.001, *t*-test with Welch correction.

*Statistical significance from apex-to-base propagation speed in patched OHCs, *p* < 0.05, *t*-test with Welch correction.

The fluorescent intensities averaged over the whole cell peaked immediately after UV flash and then decayed over time (Figure 2C, right). However, in contrast to patched OHCs, the fluorescent signal in intact OHCs decayed slower in the first ∼3 s after UV flash (Figure 2C, inset). This may be due to the saturation of Fluo-4 indicator by a larger increase of [Ca^2+^]_*i*_. Indeed, when we plot the relationship between ΔF/F_0_ values at the time point of 3 s after UV illumination versus maximal ΔF/F_0_ values in the same cell, the data from both patched and intact OHCs followed the same trend (Figure 1C). In other words, larger ΔF/F_0_ responses had a more prolonged “plateau” in Fluo-4 signal before subsequent exponential decay, which is indicative of Fluo-4 saturation.

Interestingly, the speed of base-to-apex propagation of Ca^2+^ signal (initiated by UV illumination at OHC base) was significantly faster than that of apex-to-base (initiated by UV illumination at the OHC apex) in patched OHCs (Table 2). In contrast, intact unpatched OHCs didn’t exhibit such a big difference in propagation speed between apex-to-base and base-to-apex directions (Table 2). Therefore, slow apex-to-base propagation of Ca^2+^ signal in patched OHCs results from some factors of whole-cell recordings. The most obvious factor is the constant diffusion of “fresh” (unexposed to UV illumination) NP-EGTA from the patch pipette into the cell. Since we always patched the OHCs at the base, this factor produces asymmetric Ca^2+^ buffering, thereby reducing the Ca^2+^ signal coming from the apex to the base but not affecting the propagation of free Ca^2+^ that was uncaged at the base.

### Ca^2+^-induced Ca^2+^-release at the base of OHCs

Analysis of the changes in the longitudinal intensity profile of ΔF/F_0_ Fluo-4 signal after UV illumination may reveal the sites of Ca^2+^-induced Ca^2+^ release along the OHC body. Indeed, if free Ca^2+^ is liberated by UV flash at a particulate spot within a cell, it is expected to diffuse from this uncaging spot along the cell body until fading away by Ca^2+^ extrusion mechanisms or reaching uniform steady-state increase of [Ca^2+^]_*i*_ throughout the cell. The peak of the longitudinal intensity profile of ΔF/F_0_ signal is expected to decrease throughout time but its location along the length of an OHC should stay at the original source of free Ca^2+^, i.e., the site of Ca^2+^ uncaging. This is exactly what we observed in the first ∼1.5 s after UV illumination to the apex of the patched OHCs (Figure 2D and E, red traces). However, by ∼3 s after UV illumination, the peak of ΔF/F_0_ profile suddenly shifted toward the cell nucleus and maintained there with very little signs of dissipation for the remaining ∼5 s of observation (Figure 2D, right and Figure 2E, green traces). This sudden shift of the peak in the intensity profile of Ca^2+^ signal cannot be explained by a simple diffusion. Most likely, free Ca^2+^ diffusing from the apex of the cell reached Ca^2+^-sensitive stores at the base and evoked CICR there. Then, this effect is expected to be even more prominent in the intact OHCs that would not have continuous diffusion of fresh Ca^2+^ chelator into the cell via patch pipette at the cell’s base.

Indeed, while patched OHCs with Ca^2+^ uncaging at their apex (*n* = 7) exhibited a relatively minor secondary peak at the base of the cell on 2D plots of ΔF/F_0_ profile vs. time (Figures 2D, 3A), this peak was significantly larger in intact OHCs (*n* = 13) in the same experimental conditions (Figure 3B). In a few cells that had a smaller increase of Ca^2+^ signal after uncaging, the ΔF/F_0_ signal around the nuclear region became even higher than the average value throughout the cell (data not shown). Thus, our data demonstrates the existence of Ca^2+^-induced Ca^2+^ release stores at the base of the OHCs. However, they cannot yet refute the existence of such stores at the apex of the cell because any additional signal from free Ca^2+^ released from the stores would overlap with uncaged Ca^2+^ when the UV beam is focused directly on the site of CICR. Therefore, we tried to explore a potential CICR at the OHC apex by pointing UV illumination to the base of the cell (Figure 3D, *n* = 5). We also tried fast confocal imaging (Figure 3C, *n* = 3), arguing that faster and smaller optical section imaging could help since CICR is expected to be delayed relative to UV flash. In both cases, we did not detect any secondary peaks on 2D plots of ΔF/F_0_ profile vs. time that would indicate the presence of functional CICR at the apex of OHCs. However, Ca^2+^ uncaging at the base of intact OHCs caused there a long-lasting and apparently self-sustained increase of [Ca^2+^]_*i*_ (Figure 3D). It was even possible to resolve a secondary increase in Ca^2+^ signal after dissipation of uncaged Ca^2+^ (Figure 3D, bottom). We concluded that functional Ca^2+^ stores generating CICR are located at the base but not the apex of OHCs.

### Caged IP_3_ produces Ca^2+^ release from intracellular stores throughout OHC

It has been reported that extracellular ATP can generate an increase of [Ca^2+^]_*i*_ localized at the apex of the OHC in nominal Ca^2+^-free extracellular medium, apparently by releasing Ca^2+^ from IP_3_ gated stores (Mammano et al., 1999). However, IP_3_-gated Ca^2+^ release in OHCs has not yet been visualized directly. Likewise, it is not clear whether the putative IP_3_-gated stores are functional at the OHC lateral wall and/or the base of the cell. Therefore, we imaged Ca^2+^ responses in the OHCs after IP_3_ uncaging. Since IP_3_ is available in the cytosol within milliseconds after flash photolysis, we hoped to resolve the location of the IP_3_-gated store along the length of an OHC. To separate Ca^2+^ signals in OHCs and neighboring non-sensory Deiters’ cells that are very sensitive to IP_3_ (Lagostena and Mammano, 2001), we used 100 μM of cell-impermeable caged IP_3_ supplemented with 100 μM of Fluo-4 and delivered them into an OHC through the patch pipette. As expected, IP_3_ uncaging at the OHC apex generated a very robust Ca^2+^ response (Figure 4A). However, the increase of [Ca^2+^]_*i*_ after IP_3_ uncaging was rather slow (Figure 4B), which was very different from Ca^2+^ uncaging in similar experimental conditions (Figure 1F). In average, the amplitude of IP_3_-gated increase of ΔF/F_0_ signal was 12.9 ± 1.7% and the time constant of this increase was 6.3 ± 1.0 s (*n* = 8). The uniform increase of [Ca^2+^]_*i*_ throughout an OHC was also observed when IP_3_ uncaging was performed at the base of the cell (data not shown). The most likely explanation for such a slow rise of [Ca^2+^]_*i*_ is that IP_3_ quickly diffuses from the site of uncaging throughout the cell and then stimulates slow-activating IP_3_ receptors. For comparison, we observed an order of magnitude faster Ca^2+^ responses in the supporting cells that were patched with the same intrapipette solution containing the same concentration of caged IP_3_ and stimulated by the same UV flash (Figures 4C, D). It is worth mentioning that we were able to generate Ca^2+^ responses in OHCs only with 100 μM of caged IP_3_, a significantly higher concentration than the one that is needed for IP_3_-gated Ca^2+^ release in Deiters’ or Hensen’s cells, 8 and 16 μM correspondingly (Lagostena and Mammano, 2001; Lagostena et al., 2001). We concluded that IP_3_ receptors in OHCs are less sensitive than in supporting cells, either due to the expression of a different isoform of IP_3_R or due to the downregulation of their sensitivity. As to the location of IP_3_-gated Ca^2+^ stores along the length of an OHC, our imaging does not reveal any obvious “spots” of Ca^2+^ release, suggesting that they may be distributed throughout the cell. Interestingly, simultaneous patch clamp recordings with K^+^-based intrapipette solution and −60 mV holding potential revealed a small but significant positive shift of the whole-cell current by 6.9 ± 2.8 pA (*p* < 0.05, paired *t*-test) after IP_3_ uncaging (Figure 4B, bottom), which is consistent with an outward current through Ca^2+^-activated K^+^-channels (Evans, 1996) that are located at the base of the OHCs (Oliver et al., 2000).

### Ca^2+^ uncaging does not affect prestin-based non-linear capacitance in OHCs

Our previous data suggested that OHC motor protein, prestin, could be regulated by Ca^2+^/calmodulin dependent phosphorylation (Frolenkov et al., 2000). Furthermore, subsurface cisternae, a putative intracellular Ca^2+^ store (Schulte, 1993), resides in the proximity of the OHC lateral plasma membrane, where prestin is highly expressed (Belyantseva et al., 2000; He et al., 2010; Holley and Ashmore, 1988; Holley and Ashmore, 1990; Holley et al., 1992). Therefore, a long-standing (but never proved) hypothesis suggests that the release of free Ca^2+^ from subsurface cisternae may regulate the operation of prestin-based OHC plasma membrane motors. To test this hypothesis, we explored the effects of Ca^2+^ uncaging on voltage-dependent (non-linear) capacitance of OHCs, a known “signature” of operation of prestin-based plasma membrane motors in OHCs (Santos-Sacchi, 1991). We measured the changes of OHC non-linear capacitance after establishing whole-cell recordings in the same cell before and 8.5–9.5 s after Ca^2+^ uncaging (Figure 5A). As in the above experiments (see Figure 1), the patch pipette contained Cs^+^-based intrapipette solution, supplemented with Ca^2+^-bound NP-EGTA, and Ca^2+^ indicator Fluo-4. Only the OHCs exhibiting stable recordings of whole-cell current and cell capacitance were stimulated by a UV beam, which was targeted to the upper half of the cell, away from the nucleus region that has a smaller density of plasma membrane motors compared to the later wall region of an OHC (Dallos et al., 1991; Huang and Santos-Sacchi, 1993). In all these cells, UV flash produced a very fast and substantial increase of [Ca^2+^]_*i*_ (Figure 5A, insets). Even though our hardware continuously measured OHC capacitance, we didn’t observe any noticeable changes in cell capacitance either immediately or several seconds after Ca^2+^ uncaging (Figure 5A). Statistical analysis revealed only a small rightward shift of C_*m*_(V) curve after Ca^2+^ uncaging (Figure 5B and Table 3). However, a similar rightward shift was observed in control OHCs that were stimulated by UV flash but were not loaded with NP-EGTA+Ca^2+^ and hence were not exposed to the [Ca^2+^]_*i*_ increase (Figure 5B, inset and Table 3).

**TABLE 3:** Parameters (Mean ± SE) of the Boltzmann fit for non-linear (voltage-dependent) capacitance of the same OHCs before and after Ca^2+^ uncaging (see Methods for more information on the fit).

	*C*_*lin*_ (pF)	*Q*_*max*_ (pC)	*V*_*pk*_ (mV)	*z*
**NP-EGTA+Ca^2+^+Fluo-4 (n = 14)**				
Before Ca^2+^ uncaging	4.61 ± 0.18	0.117 ± 0.027	−50.0 ± 3.3	0.84 ± 0.02
After Ca^2+^ uncaging^#^	4.61 ± 0.18	0.118 ± 0.029	−47.6 ± 3.3*	0.85 ± 0.02
**Fluo-4 only (control, n = 3)**				
Before Ca^2+^ uncaging	5.86 ± 0.09	0.309 ± 0.005	−33.8 ± 1.3	0.83 ± 0.01
After Ca^2+^ uncaging^##^	5.86 ± 0.08	0.321 ± 0.011	−30.1 ± 2.0*	0.81 ± 0.02

*Statistical significance of the changes after Ca^2+^ uncaging, *p* < 0.05, paired *t*-test.

^#^Time after Ca^2+^ uncaging: 8.5 s (9 cells), 9.5 s (5 cells), and 0.25 s (one cell).

^##^Time after Ca^2+^ uncaging: 9–11 s.

Since whole-cell configuration may result in the washout of essential diffusible molecules mediating potential Ca^2+^-dependent regulation of prestin function, we repeated these experiments in OHCs in perforated patch clamp conditions (*n* = 5). Similar to our previous observations of the larger ΔF/F_0_ changes in the intact OHCs (Figure 2C), UV uncaging produced larger Ca^2+^ changes in OHCs in perforated patch conditions (Figure 5C, bottom inset). Yet, we did not observe any statistically significant changes in the non-linear capacitance after Ca^2+^ uncaging, even in perforated patch conditions (Figure 5C). After completing these experiments, we ascertained the perforated patch conditions by breaking the plasma membrane at the tip of the pipette and monitoring the resulting drop in access resistance (Figure 5C, right inset). We concluded that the direct increase of [Ca^2+^]_*i*_ does not produce any effects on prestin-based non-linear capacitance, at least in young postnatal OHCs and on a time scale of up to 11 s.

### Raising [Ca^2+^]_*i*_ increases stereocilia bundle stiffness

Similar to the putative Ca^2+^ stores in the subsurface cisternae, the function of Ca^2+^ stores at the hair cell apex is also largely hypothetical. However, in contrast to subsurface cisternae, IP_3_-dependent Ca^2+^ release at the apex of OHCs has been observed by direct Ca^2+^ imaging in response to extracellular ATP (Mammano et al., 1999). Based on the proximity to the stereocilia bundle, it was hypothesized that the apical Ca^2+^ stores may be involved in the regulation of mechanical properties of the hair bundle (Mammano et al., 1999). This hypothesis is consistent with the old data demonstrating the increase of stereocilia bundle stiffness in chick cochlear hair cells by permeabilizing them with Ca^2+^ ionophores and raising Ca^2+^ concentration in the bath (Pae and Saunders, 1994). Therefore, our next step was to explore whether Ca^2+^ uncaging at the apex of the cell could affect the stiffness of the stereocilia bundle. To avoid confounding effects of the global Ca^2+^-induced softening of cytoskeletal stiffness, which occurs in OHCs (Frolenkov et al., 2003) and is accompanied by an increase of the fluid-jet-induced rocking movement of the cuticular plate (data not shown), we performed these experiments in the inner hair cells (IHCs). IHCs have also the advantage of larger stereocilia that are easier to monitor with automatic tracking algorithms. Using our standard intracellular solution containing NP-EGTA+Ca^2+^+Fluo-4, we established whole-cell patch clamp recording configuration, waited for at least 5 min for the intrapipette solution delivery into the cell, and then recorded MET currents and stereocilia deflections before and after UV illumination to the hair cell (Figure 6A). Although UV flash targeted to a cell through the cuticular plate was less effective in Ca^2+^ uncaging, the train of UV pulses generated a substantial increase in ΔF/F_0_ signal (Figure 6B) and a drop in whole-cell current due to activation of Ca^2+^-sensitive ion channels (Figure 6E). Simultaneous video recordings allowed off-line analysis of stereocilia bundle movements (Figure 6C) and estimating the relationship between the amplitude of hair bundle deflection (ΔX) and the fluid-jet pressure (P) in the same cell before and after UV illumination. Data showed that Ca^2+^ uncaging reduces the amplitude of fluid-jet-evoked bundle deflections, which means that the hair bundle becomes stiffer (Figure 6D). Statistical analysis demonstrated that Ca^2+^ uncaging, indeed, decrease the slope of the relationship between bundle deflection (ΔX) and fluid-jet pressure (P), which is proportional to the stereocilia bundle compliance and reciprocal to the bundle stiffness (Figure 6H). Stiffening of the stereocilia bundle was not observed in control experiments that used the same intrapipette solution but without NP-EGTA+Ca^2+^ (Figure 6H).

Interestingly, we did not observe any apparent effects of Ca^2+^ uncaging on the MET responses recorded in the same cell (Figure 6E). In a group of IHCs (*n* = 9), the average ΔF/F_0_ increase was 63 ± 6% (min/max: 34/95%). Yet, in the same cells, we did not detect any statistically significant changes in the open MET channel probability at resting bundle position (P_*OPEN*_) (Figure 6G). Amplitudes of MET currents were lined up along the same MET current-displacement curve, albeit at smaller bundle deflections after Ca^2+^ uncaging (Figure 6I). Thus, we believe that potential Ca^2+^ uncaging inside stereocilia, if present, was too small in our experiments to affect MET responses, while limited amount of liberated free Ca^2+^ from the soma of the cell cannot easily reach the tips of stereocilia. We concluded that a moderate increase of [Ca^2+^]_*i*_ within the soma of IHCs can stiffen stereocilia bundles without apparent effects on MET responses.

## Discussion

**FIGURE 7:**
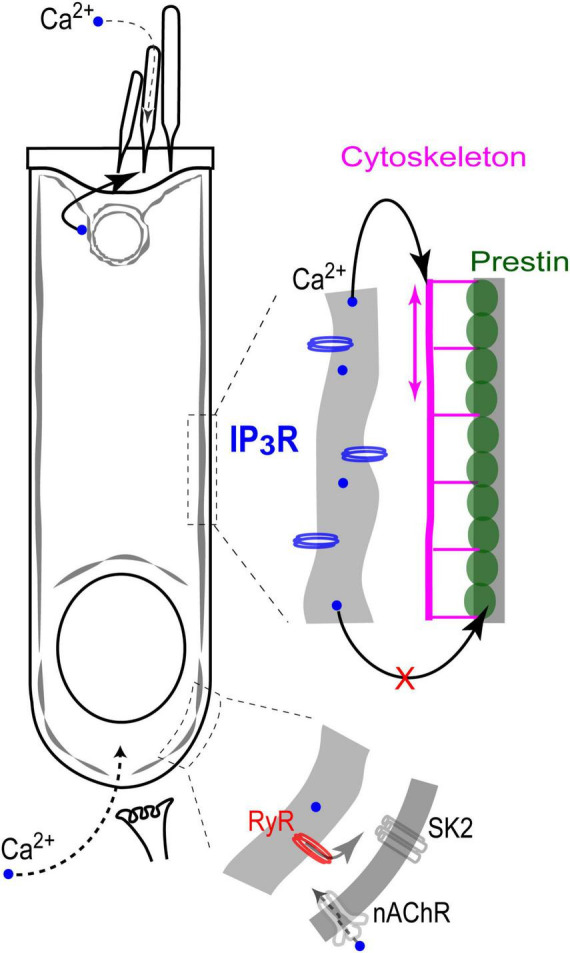
Endoplasmic reticulum Ca^2+^ stores in the cochlear OHCs. IP_3_ gated stores seem to be concentrated toward the apex of the cell and near the lateral wall. In contrast, Ca^2+^-induced Ca^2+^-release (RyR stores) dominates at the base, where it modulates ACh-mediated efferent effects on OHC intracellular potential. At the lateral wall, intracellular Ca^2+^ modulates OHC axial stiffness without affecting voltage-driven conformations of prestin. Most likely, free Ca^2+^ in this region of the cell is released from IP_3_-gated stores in subsurface cisternae. Finally, IP_3_-gated stores at the apex of the cell regulate the mechanical stiffness of the stereocilia bundle without affecting the MET apparatus (perhaps, due to the well-known sequestration of free Ca^2+^ within a stereocilium).

This is the first study that systematically explores the effects of direct uncaging of intracellular Ca^2+^ or IP_3_ in the cochlear hair cells, thus mimicking Ca^2+^ release from various intracellular Ca^2+^ stores. Our data showed evidence for: (i) Ca^2+^-induced Ca^2+^ release at the base but not apex of the OHCs; (ii) IP_3_-gated slow release of intracellular free Ca^2+^ throughout an OHC; (iii) no short-term (up to 11 s) effects of the increase in intracellular free Ca^2+^ on the operation of prestin-based OHC membrane motors; and (iv) regulation of stereocilia bundle stiffness by changes of intracellular free Ca^2+^ concentration. Although our experiments were performed in postnatal hair cells that may still develop, our data argue for different properties and different roles of spatially separated Ca^2+^ stores, which seem to be already present in hair cells at this developmental stage (Figure 7).

### Ca^2+^-induced Ca^2+^ release (CICR) from the stores at the base of OHCs

CICR has been proposed to regulate the OHC response to efferent neurotransmitter acetylcholine. This proposal was based on the experiments demonstrating that the drugs affecting either RyRs or endoplasmic Ca^2+^ ATPases also modulate acetylcholine-induced OHC hyperpolarization and/or outward K^+^ current through Ca^2+^-activated K^+^ channels (Lioudyno et al., 2004). Yet, this proposed CICR has never been visualized in OHC by Ca^2+^ imaging, perhaps due to the fact that it should occur in a narrow gap between synaptic cisternae and the plasma membrane (Fuchs, 2014). In fact, CICR in OHCs has never been demonstrated with direct Ca^2+^ imaging and, therefore, this is the first study to do this. Although our imaging experiments cannot pinpoint the exact site of CICR with nanoscale accuracy, they do show that CICR occurs at the base but not the apex of OHCs. On one hand, it is consistent with the proposed roles of CICR in the modulation of acetylcholine-induced responses in OHCs. On the other hand, it raises the question of why CICR occurs predominantly at the base of the cell and how it is related to the proposed Ca^2+^ stores in the synaptic cisternae. Post-embedding immunogold labeling with antibodies that predominantly recognize ryanodine receptor isoforms 1 (RyR1) and 2 (RyR2) revealed the highest RyR signal in the OHCs in the cytoplasmic region above the nucleus, about 30% smaller labeling at the very base of the cell, and about three-fold smaller labeling at the apex of the cell (Grant et al., 2006). These data are consistent with our imaging experiments (Figures 2, 3). However, they also indicate that the area of functional CICR in OHCs may be larger that is needed only for the modulation of acetylcholine-induced responses. The potential role of CICR in the area above the nucleus remains unknown.

### Ca^2+^ stores in the subsurface cisternae of the lateral wall of OHCs

Expression of intracellular Ca-ATPase along the lateral walls of the OHCs (Schulte, 1993; Zine and Schweitzer, 1996) provides a strong argument for the existence of the functional Ca^2+^ stores in the subsurface cisternae. However, Ca^2+^ release from these stores has never been visualized by Ca^2+^ imaging. Here, we demonstrated a slow and uniform Ca^2+^ release throughout the OHC after IP_3_ uncaging (Figure 4), suggesting that functional IP_3_-gated stores are likely to be distributed along the length of an OHC, including the lateral wall. It is consistent with our previous data showing uniform IP_3_R labeling along the lateral wall of the OHCs (Frolenkov et al., 2000). We also did not reveal any signs of CICR in the middle of an OHC, away from the nucleus region. The latter observation suggests that the lateral wall stores may be activated predominantly by IP_3_, at least in young postnatal OHCs. That said, elaborated system of the lateral wall subsurface cisternae may not be completely developed in the rodent OHCs at P12, the maximal age of our experiments (Souter et al., 1995). Therefore, functional properties of the lateral wall Ca^2+^ stores may be different in adult OHCs.

A known potential target for free Ca^2+^ released from the lateral wall subsurface cisternae is the cortical cytoskeleton that determines OHC axial stiffness. It is known that the rise of [Ca^2+^]_*i*_ causes a decrease of OHC axial stiffness (Frolenkov et al., 2003) likely mediated by Rho GTPases (Kalinec et al., 2000; Zhang et al., 2003) and downstream LIMK/cofilin-dependent actin depolymerization (Matsumoto et al., 2010). These cytoskeletal changes underlie the acetylcholine-induced changes of OHC somatic motility (Dallos et al., 1997) that occur without any changes in the operation of prestin-based plasma membrane motors (Frolenkov et al., 2000; Matsumoto et al., 2010). The experiments of this study show that even a direct increase of [Ca^2+^]_*i*_ by Ca^2+^ uncaging is not able to affect prestin motors (Figure 5). Perhaps prestin is regulated through Ca^2+^-independent mechanisms such as phosphorylation via cyclic GMP / protein kinase G (cGMP/PKG) pathway (Deak et al., 2005; Szonyi et al., 1999).

### Regulation of stereocilia bundle stiffness by IP_3_-gated stores at the apex of the hair cell

Visualization of ATP-activated Ca^2+^ release from IP_3_-dependent stores at the apex of OHCs, right beneath the cuticular plate, led to the hypothesis that these stores may be involved in the regulation of the mechanical properties of stereocilia bundle (Mammano et al., 1999). Unfortunately, we were not able to test this hypothesis directly in OHCs due to the confounding effects of Ca^2+^-dependent softening of the OHC cytoskeleton and the increased rocking motion of the cuticular plate upon fluid-jet stimulation. However, our data show that, at least in IHCs, a rise of [Ca^2+^]_*i*_ results in the stiffening of the stereocilia bundle (Figure 6). Although it is yet unclear why the increase of intracellular free Ca^2+^ produces opposite effects on somatic and hair bundle cytoskeletons (softening vs. stiffening, correspondingly), our data are consistent with the previously reported effects of Ca^2+^ on the hair bundle stiffness in chick hair cells permeabilized with ionophore (Pae and Saunders, 1994).

At first sight, it is surprising that Ca^2+^ uncaging did not affect MET responses in our experiments. However, as we already mentioned, the efficiency of UV pulses in liberating free Ca^2+^ was substantially lower when the UV beam was penetrating the cell through hair bundle and cuticular plate, perhaps, due to increased light scattering. Therefore, we do not know whether this experimental arrangement produces any sizable increase of [Ca^2+^]_*i*_ within a stereocilium—most of the visible increase in fluorescence came from within a cell (Figure 6B, insets). Second, [Ca^2+^]_*i*_ in the tips of stereocilia is expected to be in order of several micromoles (Fettiplace and Nam, 2019). It is very unlikely that our Ca^2+^ uncaging ever reached these levels, bearing in mind that we observed an increase in Fluo-4 fluorescence only within 34–95% range (Figure 6B).

The overall stiffness of a hair bundle is often separated into two components: (i) the stiffness at the stereocilia pivot points; and (ii) the stiffness associated with the tip links and the MET apparatus (Mecca et al., 2022; Tobin et al., 2019). In general, it is at least a very oversimplified assumption due to multiple other links interconnecting stereocilia, especially in OHCs (Goodyear et al., 2005). However, for the purpose of this discussion, we could separate the hair bundle stiffness into “pivot stiffness of stereocilia” and “stereocilia link-associated stiffness.” The fact that Ca^2+^ uncaging changes the hair bundle stiffness in our experiments without apparent effects on MET currents (Figure 6) may indicate that free Ca^2+^ from the soma of the cell cannot reach inside stereocilia and affects only “pivot stiffness.” It could occur through Ca^2+^-dependent modifications of proteins located specifically at the base of stereocilia (Pacentine et al., 2020). This hypothesis would be also consistent with an idea of “self-contained” Ca^2+^ compartment within a stereocilium that is generally isolated from the rest of the cell by specialized Ca^2+^ clearance mechanisms (Dumont et al., 2001; Fettiplace and Nam, 2019; Lumpkin and Hudspeth, 1998). However, we would refrain from making a definitive conclusion. Ca^2+^-induced changes in hair bundle stiffness develop within tens of seconds (Figure 6H). This slow time course allows plenty of time for any possible indirect effects, for example, by immobilizing stereocilia myosin motors and preventing them from entering stereocilia.

Independent of the potential mechanisms, our study, to the best of our knowledge, is the first to explore the changes of stereocilia bundle stiffness in cochlear hair cells in response to physiologically relevant changes of [Ca^2+^]_*i*_ inside the cell body. In the future, it would be interesting to explore whether more physiological stimuli, such as low-level extracellular ATP, could produce similar changes in stereocilia bundle stiffness.

## Data Availability

The original contributions presented in this study are included in this article/supplementary material, further inquiries can be directed to the corresponding author.
